# Age-specific association between dietary nutrient adequacy and self-rated health in Korean adolescents: a cross-sectional study based on the Korea National Health and Nutrition Examination Survey 2022–2024

**DOI:** 10.3389/fnut.2026.1880706

**Published:** 2026-08-06

**Authors:** Dongyoun Woo

**Affiliations:** Independent Researcher, Daejeon, Republic of Korea

**Keywords:** age-specific association, cross-sectional study, dietary diversity score, KNHANES, Korean adolescents, mean adequacy ratio, nutrient adequacy, self-rated health

## Abstract

**Background:**

Self-rated health (SRH) reliably predicts mortality and chronic disease risk, yet the association between the mean adequacy ratio (MAR), a nutrient adequacy index, and SRH in Korean adolescents remains underexplored.

**Methods:**

This cross-sectional study analyzed 1,053 adolescents aged 12–18 years from Korea National Health and Nutrition Examination Survey (KNHANES) 2022–2024, excluding those with extreme energy intakes for their sex and age group. MAR was calculated as the mean of nine nutrient adequacy ratios, each capped at 1.0 using the 2020 Korean Dietary Reference Intakes (KDRI). The dietary diversity score (DDS) was calculated from five food groups. SRH was dichotomized as good (1–2) or fair-to-poor (3–5), and associations were estimated using complex-sample logistic regression with a quasibinomial family.

**Results:**

In the fully adjusted model, higher MAR was associated with lower odds of fair-to-poor SRH (OR: 0.88, 95% CI: 0.78–0.99; *p* = 0.040). In exploratory analyses, this association was observed in adolescents aged 15–18 years (OR: 0.79, 95% CI: 0.68–0.93; *p* = 0.005) but not 12–14 years (OR: 1.02, 95% CI: 0.85–1.22; *p* = 0.851; age group interaction *p* = 0.017). DDS showed no association with fair-to-poor SRH (OR: 1.00, 95% CI: 0.83–1.21; *p* = 0.960). MAR was not associated with overweight (OR: 1.06, 95% CI: 0.91–1.23; *p* = 0.477) or abdominal obesity (OR: 1.03, 95% CI: 0.88–1.20; *p* = 0.737). A sensitivity analysis using the 2025 KDRI yielded consistent results: the primary association remained significant (OR: 0.87, 95% CI: 0.78–0.98; *p* = 0.027), and the age group interaction remained statistically significant (*p* = 0.013).

**Conclusion:**

Higher MAR was associated with lower odds of fair-to-poor SRH in Korean adolescents, particularly among those aged 15–18 years. Longitudinal studies are needed to confirm these findings and support nutrition programs targeting older Korean adolescents.

## 1 Introduction

Adolescence is a period of rapid physical growth and cognitive development, and nutritional status during this time lays the foundation for adult health (1). In Korea, adolescents face intense academic pressure driven by the demands of the university entrance examination system, which requires strong performance on both continuous school-based assessments and a single national college entrance examination known as Suneung, the College Scholastic Ability Test (CSAT). Many attend private tutoring academies after school, often until late in the evening. This leaves little time for proper meals, and many turn to fast food and processed options that are low in essential nutrients (2). Poor nutrition during adolescence has been linked to both immediate health consequences and long-term cardiometabolic risks (1). Understanding how nutritional status relates to the way Korean adolescents perceive their own health is therefore an important public health question.

Measured with a single question, self-rated health (SRH) reliably predicts morbidity and mortality (3, 4), and in adolescents it also reflects health-related behaviors, including dietary intake and physical activity (5, 6). Among dietary quality indices, the nutrient adequacy ratio (NAR) measures how closely each nutrient’s intake meets its recommended level. The mean adequacy ratio (MAR) averages these values across multiple nutrients, with each NAR capped at 1.0 so that adequate intake of one nutrient cannot mask deficiency in another (7). The dietary diversity score (DDS), defined as the number of food groups consumed on a given day (range 0–5) following Nakitto et al. (8), captures variety rather than nutrient adequacy. Oftedal et al. reported that better overall diet quality was associated with lower odds of fair-to-poor SRH in adults (9), yet this association has not been well studied using MAR in Korean adolescents with appropriate complex survey methods. How MAR and DDS compare as predictors of SRH in this population is also unclear, and little is known about whether the association varies by age.

This study aimed to examine the association between MAR and fair-to-poor SRH in Korean adolescents aged 12–18 years using data from the 9th KNHANES (2022–2024), and to test whether this association differed between early (12–14 years) and late (15–18 years) adolescents. DDS was analyzed in a parallel model to compare whether food group diversity independently explained the association. A sensitivity analysis using the 2025 KDRI was also conducted to examine whether the primary findings remained consistent when a more recently updated set of nutrient reference values was applied.

## 2 Materials and methods

### 2.1 Study design and data source

Data for this cross-sectional study came from the 9th Korea National Health and Nutrition Examination Survey (KNHANES). Three consecutive cycles of KNHANES (2022–2024) were pooled. Sampling weights were divided by three to account for the 3-year pooled design (pooled_wt_tot = wt_tot/3). KNHANES is a nationally representative survey conducted by the Korea Disease Control and Prevention Agency (KDCA) using a multi-stage clustered probability sampling design to represent the non-institutionalized civilian population in Korea (10). The KNHANES protocol was reviewed and approved by the Institutional Review Board of the KDCA under approval numbers 2018-01-03-4C-A (survey year 2022), 2022-11-16-R-A (survey year 2023), and 2022-11-16-R-03 (survey year 2024). Because this study is a secondary analysis of de-identified, publicly available data, written informed consent from participants was not required for the present analysis, in accordance with national legislation and institutional requirements.

### 2.2 Study participants

**Figure 1:**
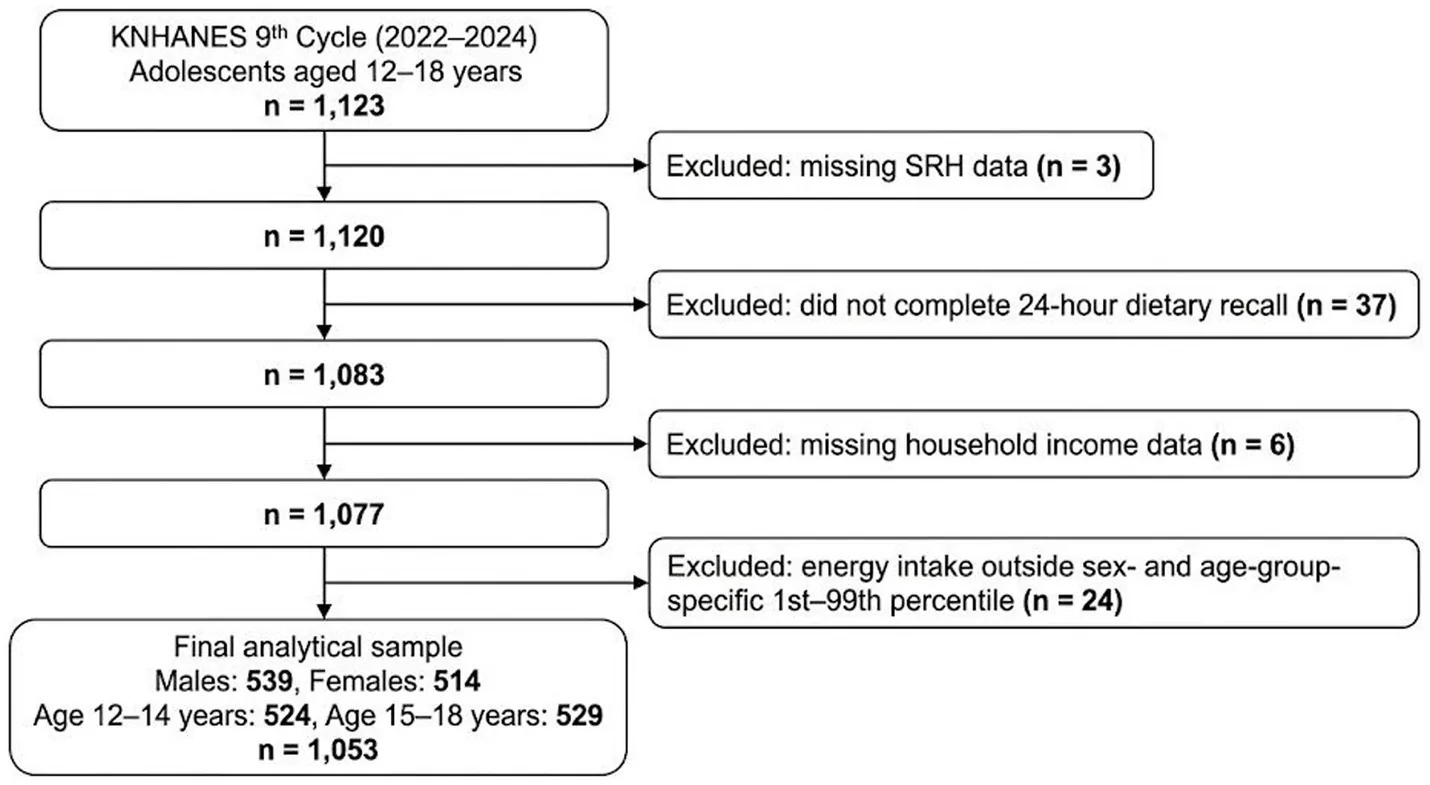
Participant selection flowchart. KNHANES, Korea National Health and Nutrition Examination Survey; SRH, self-rated health.

Adolescents aged 12–18 years from all three survey cycles were considered for inclusion, corresponding to the two adolescent age strata defined by the 2020 Korean Dietary Reference Intakes (KDRI; 12–14 and 15–18 years), which served as the reference for MAR calculation. Those who did not complete the 24-h dietary recall (24HR), had missing data on the self-rated health (SRH) question, or did not report household income were excluded. From an initial sample of 1,123 adolescents, three were excluded for missing SRH data, 37 for not completing the dietary survey, and six for missing income data. To minimize misreporting bias inherent in 24-h dietary recall, participants with energy intakes below the 1st or above the 99th percentile within subgroups defined by sex and age group were excluded as a pre-specified criterion (11). This removed an additional 24 participants, leaving a final sample of 1,053 individuals (539 males, 514 females). The full participant selection process is illustrated in Figure 1.

### 2.3 Assessment of dietary intake: MAR and DDS

Dietary intake was assessed through a single 24HR (10). The nutrient adequacy ratio (NAR) for each nutrient was calculated by dividing actual intake by the recommended nutrient intake (RNI) from the 2020 Korean Dietary Reference Intakes (KDRI) (12). Each NAR was capped at 1.0 (NAR = min[intake/RNI, 1.0]) so that high intake of one nutrient could not offset deficiency in another (7). The mean adequacy ratio (MAR) was calculated as the arithmetic mean of NARs for nine nutrients: protein, calcium, iron, vitamin A, vitamin C, thiamine, riboflavin, niacin, and dietary fiber (7, 13). Individual NAR values for each of the nine nutrients are presented in Supplementary Table S1. MAR was multiplied by 10 in all regression models so that each estimated odds ratio (OR) represents the change per 0.1-unit increase in MAR.

The dietary diversity score (DDS) was calculated by summing binary scores (0 or 1) for five food groups (cereals, meat/fish/legumes, vegetables, fruits, and dairy), giving a score from 0 to 5, following Nakitto et al. (8).

As a sensitivity analysis, MAR was recalculated using the 2025 KDRI to test whether the results would change when a more recent version of the nutrient reference values was applied (14). Results are presented in Supplementary Table S4.

### 2.4 Assessment of self-rated health

SRH was measured using the question “How would you describe your health in general?” (item D_1_1) from the KNHANES health questionnaire. Responses were on a five-point scale (1 = very good, 2 = good, 3 = fair, 4 = poor, 5 = very poor). Codes 1–2 were classified as good SRH and codes 3–5 as fair-to-poor SRH. Including “fair” in the fair-to-poor SRH category was based on prior evidence that “fair” self-rated health in adolescence carries a mortality risk similar to “poor” or “very poor” (4), and the same classification has been applied in previous Korean studies using KNHANES (15). Using only codes 4–5 as fair-to-poor SRH would have left just 69 participants (6.6%) in the outcome group, which was too small for the planned stratified analyses. Sensitivity analysis results using this alternative cut are reported in Supplementary Table S5.

### 2.5 Covariates

Sex (male/female) and age group (12–14 years/15–18 years) were included because nutritional requirements and SRH patterns may differ by sex and age group. Household income level (quintiles 1–5) was included as a socioeconomic indicator linked to food access and health behavior. Physical activity was defined as ≥60 min per day of physical activity on ≥5 days in the past week, based on KNHANES variable BE9 (code ≥6 corresponds to ≥5 days/week) (16). This was coded as a binary variable (0 = not meeting the physical activity guideline, 1 = meeting the physical activity guideline). Total energy intake (kcal/day) was included as a continuous variable. Survey year (2022, 2023, 2024) was included as a categorical variable to control for time trends across cycles.

Overweight was defined as BMI at or above the 85th percentile for age and sex, based on the 2017 Korean National Growth Charts (17). Abdominal obesity was defined as waist circumference at or above the 90th percentile using sex- and age-specific cutoffs from Lee et al. (18). Both variables were excluded from the main models because they may be mediators on the pathway from MAR to SRH. In other words, better nutrient adequacy may be partly associated with lower odds of fair-to-poor SRH through its relationship with body weight, and including these variables in the model would block that pathway and underestimate the total association. They were analyzed separately as secondary outcomes, with results reported in Supplementary Table S3.

### 2.6 Statistical analysis

All statistical analyses were performed using R version 4.5.3 (2026-03-11; R Foundation for Statistical Computing, Vienna, Austria) with the survey package (19). R code for the statistical analysis was developed with assistance from Claude Sonnet 4.6 (Anthropic, San Francisco, CA, USA), an AI language model; all code was reviewed and verified by the authors before use. Figure 1 was prepared with the assistance of Gemini 3.1 Pro (Google, Mountain View, CA, USA), an AI-based image generation tool; the output was reviewed and verified by the author.

Complex survey methods were applied to account for the KNHANES stratified, multistage sampling design (stratification variable: kstrata; clustering unit: psu). Sampling weights were divided by three (pooled_wt_tot = wt_tot/3) to maintain national representativeness for the pooled 3-year dataset. The option “survey.lonely.psu” was set to “adjust” to handle strata with a single primary sampling unit.

Participant characteristics are presented separately for the good SRH and fair-to-poor SRH groups (Table 1). Continuous variables are shown as weighted mean (standard error) and categorical variables as weighted proportion (%). Group differences were tested using complex-sample linear regression (svyglm) for continuous variables and the complex-sample *χ*^2^ test (svychisq) for categorical variables.

**Table 1:** Weighted characteristics of the study participants by self-rated health group (KNHANES 2022–2024; *n* = 1,053).

Variable	Total (*n* = 1,053)	Good SRH (*n* = 587)	Fair-to-poor SRH (*n* = 466)	*p*-value
Sociodemographic				
Age group (12–14 years), %	40.7	45.4	35.1	0.001
Sex (male), %	51.6	54.4	48.3	0.063
Household income quintile, %	–	–	–	0.302
Q1 (lowest)	19.1	18.1	20.5	
Q2	19.8	18.9	20.9	
Q3	19.0	21.1	16.5	
Q4	21.6	20.4	23.1	
Q5 (highest)	20.4	21.6	19.0	
Lifestyle				
Physical activity (≥5 d/week), %	8.8	11.1	6.1	0.005
Total energy intake (kcal/day), mean (SE)	1,908 (24.5)	1,980 (34.1)	1,822 (39.3)	0.004
Dietary exposure				
MAR, mean (SE)	0.659 (0.006)	0.679 (0.007)	0.634 (0.011)	0.001
DDS, mean (SE)	4.267 (0.027)	4.284 (0.033)	4.247 (0.042)	0.480
Anthropometric				
Overweight, %^1^	23.4	18.0	29.8	<0.001
Abdominal obesity, %^2^	20.1	15.0	26.1	<0.001

Values are weighted proportions (%) or weighted means (SE). All analyses incorporated complex survey weights, stratification, and clustering variables. Group differences were tested using complex-sample linear regression (svyglm) for continuous variables and the complex-sample *χ*^2^ test (svychisq) for categorical variables. MAR: mean adequacy ratio (range 0–1; nine nutrients, 2020 KDRI); DDS: dietary diversity score (range 0–5 food groups); SE: standard error.

1 Overweight: BMI ≥ 85th percentile for age and sex (Kim et al. (17); available in *n* = 1,048).

2 Abdominal obesity: waist circumference ≥ 90th percentile for sex and age group (Lee et al. (18); available in *n* = 1,018).

The associations of MAR (×10; per 0.1-unit increase) and DDS with fair-to-poor SRH were estimated as ORs and 95% confidence intervals (CIs) using complex survey logistic regression with a quasibinomial family. The quasibinomial family was used to account for over-dispersion that arises in complex survey data when a standard binomial model may underestimate variance. Three models with increasing levels of adjustment were built: Model 1 (sex, age group, survey year); Model 2 (Model 1 plus household income, physical activity); and Model 3 (Model 2 plus total energy intake; fully adjusted model). Results are presented in Table 2. To examine potential residual confounding by variables not included in the primary models, four additional sensitivity analyses were conducted by separately adding the following covariates to the fully adjusted model: depressive symptoms (BP5, 2-week depressive episode, yes/no), daily sitting time (BE8_1, h/day), perceived stress level (BP1, high vs. low), and dietary supplement use (LS_1YR, use of supplements for ≥2 weeks in the past year, yes/no). These variables were selected because they were consistently collected across all three KNHANES cycles (2022–2024) and applicable to adolescent respondents.

**Table 2:** Odds ratios (ORs) and 95% confidence intervals (CIs) for fair-to-poor self-rated health per 0.1-unit increase in MAR and per 1-unit increase in DDS.

Predictor	Model 1^1^	Model 2^2^	Model 3^3^
MAR (per 0.1-unit increase)			
OR (95% CI)	0.88 (0.81–0.96)	0.88 (0.81–0.96)	0.88 (0.78–0.99)
*p*-value	0.003	0.005	0.040
DDS (per 1-unit increase)			
OR (95% CI)	0.97 (0.81–1.15)	0.97 (0.81–1.17)	1.00 (0.83–1.21)
*p*-value	0.700	0.776	0.960

Fair-to-poor SRH was defined as response codes 3–5 on a five-point scale (“fair,” “poor,” “very poor”; good SRH = codes 1–2). All analyses incorporated complex survey weights with a quasibinomial family to account for overdispersion. For all models, *n* = 1,053. MAR was multiplied by 10 so that each OR represents the change per 0.1-unit increase in MAR.

1 Model 1: adjusted for sex, age group, and survey year.

2 Model 2: Model 1 plus household income quintile and physical activity.

3 Model 3 (fully adjusted model): Model 2 plus total energy intake.

MAR and DDS were analyzed in separate parallel models (MAR replaced by DDS in the DDS model); no model included both simultaneously.

Stratified analyses by sex and age group were performed, and multiplicative interaction terms (MAR × sex, MAR × age group) were tested using regTermTest() in the R survey package (Table 3). These interaction analyses were exploratory, as they were not pre-specified before data collection. A four-group exploratory subgroup analysis (males 12–14, males 15–18, females 12–14, females 15–18) and a three-way interaction test (MAR × sex × age group) were also conducted (Supplementary Table S2). No formal adjustment for multiple comparisons was applied.

**Table 3:** Odds ratios (ORs) and 95% CIs for fair-to-poor self-rated health per 0.1-unit increase in MAR, stratified by age group and sex (Model 3).

Subgroup	*n*	OR (95% CI)	*p*-value	*p* for interaction^1^
Age group				0.017
12–14 years	524	1.02 (0.85–1.22)	0.851	
15–18 years	529	0.79 (0.68–0.93)	0.005	
Sex				0.090
Male	539	0.94 (0.78–1.12)	0.477	
Female	514	0.80 (0.68–0.95)	0.009	

Model 3 covariates: sex (or age group, as appropriate for each stratum), survey year, household income quintile, physical activity, and total energy intake. All analyses incorporated complex survey weights, stratification, and clustering variables. Unweighted sample sizes are shown as *n*.

1 Interaction *p*-values were obtained using regTermTest() in the R survey package. These interaction analyses were exploratory and were not pre-specified before data collection. No formal adjustment for multiple comparisons was applied.

As secondary outcomes, the associations of MAR with overweight and abdominal obesity were analyzed using the same survey-weighted logistic regression approach (Supplementary Table S3). Statistical significance was set at a two-sided *p* < 0.05.

## 3 Results

### 3.1 Participant characteristics

The general characteristics of the 1,053 participants are shown in Table 1. The good SRH group comprised 587 individuals and the fair-to-poor SRH group comprised 466 individuals, with a weighted prevalence of fair-to-poor SRH of 45.2%. The weighted mean MAR was significantly lower in the fair-to-poor SRH group (0.634, SE = 0.011) than in the good SRH group (0.679, SE = 0.007; *p* = 0.001), while DDS did not differ significantly between groups (4.284 vs. 4.247; *p* = 0.480). Overweight was more prevalent in the fair-to-poor SRH group (29.8%) than in the good SRH group (18.0%; *p* < 0.001), and abdominal obesity was also more prevalent in the fair-to-poor SRH group (26.1%) than in the good SRH group (15.0%; *p* < 0.001). Mean energy intake was lower in the fair-to-poor SRH group (1,822 kcal/day, SE = 39.3) than in the good SRH group (1,980 kcal/day, SE = 34.1; *p* = 0.004). The proportion of adolescents aged 12–14 years was lower in the fair-to-poor SRH group (35.1%) than in the good SRH group (45.4%; *p* = 0.001). Meeting the physical activity guideline was also less common in the fair-to-poor SRH group (6.1% vs. 11.1%; *p* = 0.005). Sex (*p* = 0.063) and household income level (*p* = 0.302) did not differ significantly between groups. NAR values for each of the nine nutrients by SRH group are presented in Supplementary Table S1.

### 3.2 Association between MAR and self-rated health

Table 2 presents the ORs for fair-to-poor SRH associated with MAR across the three adjustment models. MAR showed a significant inverse association with fair-to-poor SRH in all three models. In the fully adjusted model (Model 3), each 0.1-unit increase in MAR was associated with lower odds of fair-to-poor SRH (OR: 0.88, 95% CI: 0.78–0.99; *p* = 0.040). The association was consistent in Model 1 (OR: 0.88, 95% CI: 0.81–0.96; *p* = 0.003) and Model 2 (OR: 0.88, 95% CI: 0.81–0.96; *p* = 0.005).

### 3.3 Association between DDS and self-rated health

DDS was not significantly associated with fair-to-poor SRH in any of the three adjustment models (Model 1: OR: 0.97, 95% CI: 0.81–1.15, *p* = 0.700; Model 2: OR: 0.97, 95% CI: 0.81–1.17, *p* = 0.776; Model 3: OR: 1.00, 95% CI: 0.83–1.21, *p* = 0.960; Table 2).

### 3.4 Stratified analyses by age group and sex

Table 3 presents ORs for fair-to-poor SRH associated with MAR, stratified by age group and sex. The inverse association was significant only among adolescents aged 15–18 years (OR: 0.79, 95% CI: 0.68–0.93; *p* = 0.005) and was not observed among those aged 12–14 years (OR: 1.02, 95% CI: 0.85–1.22; *p* = 0.851). The age group interaction was statistically significant in this exploratory analysis (*p* for interaction = 0.017). The sex interaction did not reach statistical significance (*p* for interaction = 0.090). Descriptively, an inverse association was observed in females (OR: 0.80, 95% CI: 0.68–0.95; *p* = 0.009) but not in males (OR: 0.94, 95% CI: 0.78–1.12; *p* = 0.477); this apparent sex-specific pattern should be interpreted with caution given the non-significant interaction term.

### 3.5 Nutrient-level patterns

Individual NAR values are presented in Supplementary Table S1. Seven of the nine nutrients showed significantly lower NARs in the fair-to-poor SRH group, including protein (0.929 vs. 0.876; *p* < 0.001), calcium (0.543 vs. 0.499; *p* = 0.013), vitamin A (0.490 vs. 0.448; *p* = 0.033), riboflavin (0.873 vs. 0.830; *p* = 0.008), niacin (0.727 vs. 0.675; *p* = 0.006), dietary fiber (0.642 vs. 0.601; *p* = 0.013), and thiamine (0.833 vs. 0.755; *p* < 0.001). The largest between-group gap was observed for thiamine, at 0.078 NAR units. Iron showed no significant between-group difference (0.554 vs. 0.528; *p* = 0.157); approximately 90% of participants in both groups had inadequate iron intake (good SRH: 90.9%, fair-to-poor SRH: 90.8%), indicating a floor effect with limited discriminative power between groups. For niacin, the proportion with inadequate intake was nearly identical between groups (74.0% vs. 77.0%) despite the statistically significant between-group difference in mean NAR (0.727 vs. 0.675; *p* = 0.006). Vitamin C was the only nutrient for which the direction reversed: a higher proportion with inadequate intake was found in the good SRH group (80.9% vs. 77.4%), though the difference was not significant (*p* = 0.283).

### 3.6 Exploratory subgroup analysis

In an exploratory subgroup analysis not pre-specified before data collection, the inverse association between MAR and fair-to-poor SRH was strongest in females aged 15–18 years (OR: 0.70, 95% CI: 0.56–0.88; *p* = 0.002; Supplementary Table S2), though this finding should be cautiously interpreted given that the three-way interaction (MAR × sex × age group) was not significant (*p* = 0.238). No significant association was found in the other three subgroups (males aged 12–14 years, females aged 12–14 years, or males aged 15–18 years). No formal adjustment for multiple comparisons was applied, as these analyses were exploratory.

### 3.7 Secondary outcomes and sensitivity analysis

MAR was not significantly associated with overweight (OR: 1.06, 95% CI: 0.91–1.23; *p* = 0.477) or abdominal obesity (OR: 1.03, 95% CI: 0.88–1.20; *p* = 0.737) in the fully adjusted models (Supplementary Table S3). The sensitivity analysis using the 2025 KDRI yielded consistent results: the inverse association between MAR and fair-to-poor SRH remained significant (OR: 0.87, 95% CI: 0.78–0.98; *p* = 0.027), and the age group interaction remained statistically significant in this exploratory analysis (*p* for interaction = 0.013; Supplementary Table S4). The sensitivity analysis using the stricter SRH cut (codes 4–5 only as poor SRH, excluding code 3; *n* = 69, 6.6%) did not reach statistical significance overall (OR: 0.81, 95% CI: 0.64–1.01; *p* = 0.066), likely reflecting reduced statistical power given the small outcome group. The direction of association was preserved, and the association remained significant among adolescents aged 15–18 years (OR: 0.74, 95% CI: 0.57–0.97; *p* = 0.030) but not among those aged 12–14 years (OR: 0.95, 95% CI: 0.67–1.35; *p* = 0.775; Supplementary Table S5).

To examine potential residual confounding by psychological and behavioral factors, four additional sensitivity models were estimated using variables consistently collected across all three KNHANES cycles. First, depressive symptoms (BP5: 2-week depressive episode, yes/no) were added to the model. Depressive symptoms were associated with higher odds of fair-to-poor SRH (OR = 2.04, 95% CI 1.21–3.43, *p* = 0.008), and the MAR–SRH association was largely unchanged (OR = 0.880, 95% CI 0.777–0.996, *p* = 0.044). Second, daily sitting time (BE8_1, averaging 11.4 h/day) was additionally adjusted for. Each additional hour of sitting was associated with higher odds of fair-to-poor SRH (OR = 1.08, 95% CI 1.02–1.14, *p* = 0.007), and the MAR–SRH association remained largely unchanged (OR = 0.882, 95% CI 0.779–0.999, *p* = 0.048). Third, perceived stress (BP1) was added to the model. High perceived stress was associated with considerably higher odds of fair-to-poor SRH (OR = 2.17, 95% CI 1.55–3.05, *p* < 0.001), and the MAR–SRH association was similar in direction and magnitude, and slightly stronger, after this adjustment (OR = 0.872, 95% CI 0.769–0.989, *p* = 0.033). Additionally, dietary supplement use (LS_1YR: use of supplements for ≥2 weeks in the past year) was included as a covariate. Supplement use was not associated with fair-to-poor SRH (OR = 1.00, 95% CI 0.75–1.33, *p* = 0.995), and the MAR–SRH association was largely unchanged (OR = 0.878, 95% CI 0.776–0.995, *p* = 0.041). Across all four sensitivity models, the primary association was consistent with the main analysis.

## 4 Discussion

### 4.1 Summary of Main findings

This study found that higher MAR was associated with lower odds of fair-to-poor SRH in Korean adolescents aged 12–18 years (OR: 0.88, 95% CI: 0.78–0.99; *p* = 0.040), after full adjustment for sex, age group, household income, physical activity, and total energy intake (Table 2). In exploratory stratified analyses, this inverse association was significant among adolescents aged 15–18 years (OR: 0.79; *p* = 0.005) but not among those aged 12–14 years (OR: 1.02; *p* = 0.851), with a statistically significant age group interaction (*p* = 0.017; Table 3). DDS was not significantly associated with fair-to-poor SRH (OR: 1.00; *p* = 0.960; Table 2). Because MAR and DDS were analyzed in separate parallel models rather than directly compared within a single model, this pattern should not be read as formal evidence that nutrient adequacy is a stronger predictor than food group diversity; it instead suggests that meeting individual nutrient targets, rather than food group variety alone, was associated with self-perceived health in this sample. Oftedal et al. reported a similar directional pattern in Australian adults, with poor diet quality prospectively associated with incident fair-to-poor SRH (OR: 1.38) (9); comparable associations between dietary quality and self-perceived health have also been observed in adolescent samples from other countries (5, 20). The present findings extend this evidence to Korean adolescents using nationally representative data and a nutrient-level composite measure.

### 4.2 Nutrient-level patterns

Several nutrient-level findings support plausible biological links (Supplementary Table S1). Thiamine showed the largest between-group NAR gap (0.078 units). Thiamine is an essential coenzyme in carbohydrate metabolism and ATP production, and poor thiamine status has been associated with fatigue and reduced physical performance (21), symptoms that may be associated with lower self-perceived health. Protein showed the second-largest gap (0.053 units), and adequate protein intake supports growth, muscle synthesis, and cognitive development in adolescents (22). Riboflavin, niacin, and dietary fiber also showed lower NARs in the fair-to-poor SRH group, and these nutrients support energy metabolism and gut function (1, 23). Seven of the nine nutrients showed significantly lower NARs in the fair-to-poor SRH group (all *p* < 0.05; Supplementary Table S1), with the exception of iron and vitamin C, suggesting that the observed association may reflect a distributed pattern of nutritional inadequacy rather than a deficiency in any single nutrient.

### 4.3 Heterogeneous patterns at the nutrient level

Not all between-group NAR differences were statistically significant, and several nutrients showed patterns worth examining further (Supplementary Table S1). Vitamin C was the only nutrient showing a reversal in inadequacy rate, despite mean NAR following the expected pattern (0.521 vs. 0.496): the proportion with inadequate intake was slightly higher in the good SRH group (80.9% vs. 77.4%), though the difference was not significant (*p* = 0.283). Korean adolescents with fair-to-poor SRH may more frequently consume energy and health drinks to cope with fatigue from academic demands (24, 25), potentially explaining the paradoxically higher vitamin C intake observed in this group; this hypothesis requires direct investigation. A different pattern was observed for both niacin and dietary fiber: mean NAR was significantly lower in the fair-to-poor SRH group for both nutrients (niacin *p* = 0.006; dietary fiber *p* = 0.013), yet inadequacy rates were nearly identical between groups (niacin: 74.0% vs. 77.0%; dietary fiber: 85.8% vs. 87.0%). This suggests that the between-group differences may be concentrated among those with higher but still below-reference intakes, a distinction that a binary adequacy threshold cannot detect.

### 4.4 Age interaction: exploratory

The age group interaction (*p* = 0.017) was found in exploratory analyses that were not pre-specified, and should be interpreted with caution (Table 3). Among adolescents aged 12–14 years, dietary intake largely reflects closer parental oversight (1), including dinner at home, which may reduce reliance on processed or fast food and support more adequate nutrient intake. Younger adolescents may also be relatively less able to link physical symptoms to dietary patterns, widening the gap between nutritional status and self-perceived health.

Among those aged 15–18 years, dietary autonomy increases alongside academic demands; preparation for the national college entrance examination (Suneung, the College Scholastic Ability Test) extends daily schedules (26), leaving less time for regular meals. Many Korean high schools operate evening self-study sessions that extend the school day until late at night, during which schools typically provide supervised dinners. Students who attend private tutoring academies after school, by contrast, receive no equivalent meal support and may depend on eating out, fast food, or delivery meals. Rising nutritional requirements during late adolescence may also increase susceptibility to the perceived health effects of poor nutrient intake (27). The strongest exploratory association appeared in females aged 15–18 years (OR: 0.70; *p* = 0.002; Supplementary Table S2), which could cautiously be consistent with the effects of menstrual iron losses and dietary restriction related to weight concerns common in this subgroup (6, 27). However, the three-way interaction (MAR × age group × sex) was not significant (*p* = 0.238), and this subgroup finding should be interpreted at a hypothesis-generating level.

### 4.5 Null association with adiposity and sensitivity analysis using the 2025 KDRI

MAR was not significantly associated with overweight (OR: 1.06, 95% CI: 0.91–1.23; *p* = 0.477) or abdominal obesity (OR: 1.03, 95% CI: 0.88–1.20; *p* = 0.737) in the fully adjusted models (Supplementary Table S3). Given the wide confidence intervals, this null finding should not be read as evidence that the MAR–SRH association is independent of adiposity; it may instead reflect limited statistical power to detect a true effect. In a separate sensitivity analysis, substituting the 2025 KDRI reference values for the primary 2020 KDRI yielded consistent results (OR: 0.87, 95% CI: 0.78–0.98; *p* = 0.027), with the age group interaction remaining significant (*p* for interaction = 0.013; Supplementary Table S4), further supporting the consistency of the primary findings.

### 4.6 Theoretical implications and practical suggestions

These findings point to several theoretical implications. The present findings add to a body of prior work reporting associations between dietary quality and SRH in Western populations (5, 9), yet evidence specifically linking nutrient adequacy to SRH in Korean adolescents has been largely absent. The present findings suggest that higher nutrient adequacy was associated with lower odds of fair-to-poor SRH in Korean adolescents. MAR, but not DDS, was associated with self-rated health in the present analyses. However, because the two indices capture different dimensions of diet quality and were not formally compared, no conclusions can be drawn regarding their relative predictive value. The significant age group interaction (Table 3) suggests that the association between nutrient adequacy and SRH may be particularly relevant among older adolescents aged 15–18 years, a pattern that has received little attention in prior work on adolescent dietary health. To illustrate the magnitude of the association on the odds scale, adolescents at the 75th percentile of MAR (0.788) had an estimated 27% lower odds of fair-to-poor SRH than those at the 25th percentile (0.547), based on the fully adjusted OR (0.88^2.41^ ≈ 0.73).

Practically, older adolescents aged 15–18 years represent a group warranting targeted monitoring and hypothesis-driven intervention research. School nutrition teachers could incorporate nutrient adequacy monitoring, with attention to thiamine, protein, and niacin (the nutrients showing the largest NAR mean differences between Good and Fair-to-poor SRH groups in Supplementary Table S1), alongside food variety assessments. This monitoring could inform both nutrition education programs for students and meal menu adjustments by school nutrition teachers to better meet specific nutrient targets. At the policy level, extending structured meal provision to evening self-study sessions may help students who currently rely on eating out, fast food, or delivery meals after school (28), where nutrient quality may be harder to control. Public health dietary guidelines for Korean adolescents could go beyond general food-group recommendations to address specific nutritional shortfalls, particularly thiamine, riboflavin, and protein.

### 4.7 Limitations

This study has several limitations. First, this was a cross-sectional study; reverse causation cannot be excluded, as it is not possible to determine whether low MAR preceded fair-to-poor SRH or whether adolescents who already reported fair-to-poor SRH were also eating less well. Because of the cross-sectional design, causal inference cannot be drawn from these findings, and the results should be interpreted as evidence of association only. Second, dietary intake was assessed from a single 24-h dietary recall, which may not represent habitual intake due to within-person variability and systematic underreporting (29, 30). Within-person variability in a single-day assessment would attenuate the observed associations toward the null, and the observed association may represent a conservative estimate of the true MAR–SRH relationship. Future studies using repeated recalls or biomarker measures of nutritional status would provide more stable exposure estimates (31). Third, the exploratory subgroup analyses were conducted without formal adjustment for multiple comparisons. As shown in Supplementary Table S2, four sex-by-age subgroups were tested without correction for multiple comparisons, so the probability of at least one false positive increases. These findings should therefore be treated as hypothesis-generating rather than confirmatory. Fourth, DDS derived from a single 24-h recall may be less sensitive than composite dietary quality indices as a measure of overall diet quality in this population. Fifth, the study population was restricted to adolescents aged 12–18 years, and whether similar associations exist in younger children remains an open question, as the SRH self-report item may not be reliably interpreted below age 12. Future research should also consider adolescents with intellectual or developmental disabilities. These groups are often underserved by existing nutrition programs yet may benefit the most from targeted nutritional support, and developing assessment approaches suited to adolescents who have difficulty perceiving or communicating their own health status would be an important step toward more equitable nutrition intervention. Future longitudinal studies examining how MAR and SRH change together over time would help clarify whether the association between the two is causal and, if so, in which direction. Similar studies in other East Asian adolescent populations, where rice-based dietary patterns and high-pressure examination systems are comparable to those in Korea, would help clarify whether the age-specific pattern found here is unique to Korea or more broadly shared across the region. Sixth, the 24-h dietary recall does not capture nutrient contributions from dietary supplements; the LS_1YR variable available in KNHANES records only binary supplement use and does not provide information on supplement type, dosage, or nutrient content, so the contribution of supplements to actual nutrient adequacy remains unquantified. Finally, the vitamin C reversal in Supplementary Table S1 calls for direct investigation: the good SRH group had a higher rate of intake below the KDRI reference value (80.9%) than the fair-to-poor SRH group (77.4%), contrary to expectation, and the mechanism behind this pattern remains unclear.

Despite these limitations, the present study has several strengths. The data came from a large, nationally representative sample of Korean adolescents, and complex survey weights were applied throughout to account for the KNHANES complex survey design. The association between MAR and SRH in Korean adolescents has not been well studied, and these findings may support prioritizing nutrient adequacy monitoring and targeted dietary support for Korean high school students aged 15–18 years, whose longer school hours and reliance on eating out make it harder to maintain balanced meals. Future longitudinal studies following Korean adolescents from middle school into high school could help clarify whether changes in nutrient adequacy predict changes in self-perceived health.

## Data Availability

Publicly available datasets were analyzed in this study. This data can be found here: Repository: Korea National Health and Nutrition Examination Survey (KNHANES) Link: https://knhanes.kdca.go.kr/knhanes/main.do.
