# Supplementary material for: Age-specific association between dietary nutrient adequacy and self-rated health in Korean adolescents: a cross-sectional study based on the Korea National Health and Nutrition Examination Survey 2022–2024
Source: Front Nutr. 2026 Aug 6;13:1880706. doi: 10.3389/fnut.2026.1880706 (PMC13489852; doi:10.3389/fnut.2026.1880706)
Supplement: Supplementary file 1 [file Table_1.DOCX]

Supplementary Material

# 1 Supplementary Tables

## 1.1 Supplementary Table S1. Weighted mean nutrient adequacy ratios (NARs) and proportions with inadequate intake by self-rated health group (KNHANES 2022–2024; n = 1,053).

| Nutrient | Total NAR, mean (SE) | NAR,  Good SRH | NAR,  Fair-to-poor SRH | Inadequate, Good SRH (%) | Inadequate, Fair-to-poor SRH (%) | p value |
| --- | --- | --- | --- | --- | --- | --- |
| Protein | 0.905 (0.006) | 0.929 | 0.876 | 30.4 | 41.6 | <0.001 |
| Calcium | 0.523 (0.009) | 0.543 | 0.499 | 90.8 | 94.5 | 0.013 |
| Iron | 0.542 (0.008) | 0.554 | 0.528 | 90.9 | 90.8 | 0.157 |
| Vitamin A | 0.471 (0.010) | 0.490 | 0.448 | 90.7 | 92.5 | 0.033 |
| Vitamin C | 0.510 (0.012) | 0.521 | 0.496 | 80.9 | 77.4 | 0.283 |
| Thiamine (B1) | 0.798 (0.009) | 0.833 | 0.755 | 51.9 | 63.7 | <0.001 |
| Riboflavin (B2) | 0.853 (0.008) | 0.873 | 0.830 | 44.5 | 50.7 | 0.008 |
| Niacin | 0.704 (0.009) | 0.727 | 0.675 | 74.0 | 77.0 | 0.006 |
| Dietary fiber | 0.623 (0.009) | 0.642 | 0.601 | 85.8 | 87.0 | 0.013 |

NAR: nutrient adequacy ratio (range 0–1; capped at 1.0 per nutrient). Inadequate intake was defined as NAR < 1.0 (i.e., intake below the recommended nutrient intake [RNI] from the 2020 KDRI). p values are from complex-sample linear regression (svyglm).

## 1.2 Supplementary Table S2. Exploratory subgroup analysis of the association between MAR (per 0.1-unit increase) and fair-to-poor SRH, stratified by sex (male/female) × age group (12–14/15–18 years) – four subgroups (Model 3).

| Subgroup | n | OR (95% CI) | p value |
| --- | --- | --- | --- |
| Males, 12–14 years | 264 | 1.05 (0.80–1.38) | 0.704 |
| Males, 15–18 years | 275 | 0.87 (0.69–1.09) | 0.228 |
| Females, 12–14 years | 260 | 0.98 (0.75–1.27) | 0.867 |
| Females, 15–18 years | 254 | 0.70 (0.56–0.88) | 0.002 |
| Three-way interaction (MAR × sex × age group) | 1,053 | – | 0.238 |

Model 3 covariates: sex, age group, survey year, household income quintile, physical activity, and total energy intake. These analyses were not pre-specified. No formal adjustment for multiple comparisons was applied. Results should be treated as hypothesis-generating only.

## 1.3 Supplementary Table S3. Association of MAR (per 0.1-unit increase) with overweight and abdominal obesity (Model 3).

| Outcome | n | OR (95% CI) | p value |
| --- | --- | --- | --- |
| Overweight ¹ | 1,048 | 1.06 (0.91–1.23) | 0.477 |
| Abdominal obesity ² | 1,018 | 1.03 (0.88–1.20) | 0.737 |

Model 3 covariates: sex, age group, survey year, household income quintile, physical activity, and total energy intake.

¹ Overweight: BMI ≥ 85th percentile for age and sex.

² Abdominal obesity: waist circumference ≥ 90th percentile for sex and age group.

## 1.4 Supplementary Table S4. Sensitivity analysis using the 2025 KDRI: ORs for fair-to-poor SRH per 0.1-unit increase in MAR.

| Model / Subgroup | OR (95% CI) | p value |
| --- | --- | --- |
| Model 3 (fully adjusted) | 0.87 (0.78–0.98) | 0.027 |
| Age 12–14 years | 1.02 (0.86–1.21) | 0.825 |
| Age 15–18 years | 0.79 (0.67–0.92) | 0.003 |
| p for age group interaction | – | 0.013 |

MAR was recalculated using 2025 KDRI reference values; updated reference values were applied for calcium, iron, niacin, and dietary fiber. Model 3 covariates identical to main analysis.

## 1.5 Supplementary Table S5. Sensitivity analysis using the alternative SRH dichotomization (codes 4–5 as poor SRH): ORs for poor SRH per 0.1-unit increase in MAR (Model 3).

| Model / Subgroup | n (Poor SRH) | OR (95% CI) | p value |
| --- | --- | --- | --- |
| Model 3 (fully adjusted) | 69 (6.6%) | 0.81 (0.64–1.01) | 0.066 |
| Age 12–14 years | 27 | 0.95 (0.67–1.35) | 0.775 |
| Age 15–18 years | 42 | 0.74 (0.57–0.97) | 0.030 |

Poor SRH was defined as response codes 4–5 ("poor" and "very poor") on the five-point scale (Good = codes 1–2–3). The main analysis defined fair-to-poor SRH as codes 3–5 (n = 466; 45.2%). The smaller outcome group (n = 69) may have reduced statistical power; the non-significant overall association (p = 0.066) should therefore be interpreted with caution. The direction of association and the age-specific pattern (Age 15–18 years significant: p = 0.030) were consistent with the main analysis. Model 3 covariates: sex, age group, survey year, household income quintile, physical activity, and total energy intake.
